# Cost of oral cholera vaccine delivery in a mass immunization program for children in urban Bangladesh

**DOI:** 10.1016/j.jvacx.2022.100247

**Published:** 2022-12-05

**Authors:** Abdur Razzaque Sarker, Ashraful Islam Khan, Md. Taufiqul Islam, Fahima Chowdhury, Farhana Khanam, Sophie Kang, Faisal Ahmmed, Justin Im, Deok Ryun Kim, Birkneh Tilahun Tadesse, Tasnuva Ahmed, Asma Binte Aziz, Masuma Hoque, Juyeon Park, Xinxue Liu, Gideok Pak, Khalequ Zaman, Florian Marks, Jerome H. Kim, John D. Clemens, Firdausi Qadri

**Affiliations:** a Population Studies Division, Bangladesh Institute of Development Studies (BIDS), Bangladesh; b International Centre for Diarrheal Disease Research, Bangladesh (icddr,b), Dhaka, Bangladesh; c International Vaccine Institute, Seoul, Republic of Korea; d Division of Clinical Pharmacology, Department of Laboratory Medicine, Karolinska Institutet, Karolinska University Hospital Huddinge, 14186 Stockholm, Sweden; e Center for Innovative Drug Development and Therapeutic Trials for Africa, College of Health Sciences, Addis Ababa University, Addis Ababa P.O. Box 9086, Ethiopia; f Cambridge Institute of Therapeutic Immunology and Infectious Disease, University of Cambridge School of Clinical Medicine, Cambridge Biomedical Campus, Cambridge CB2 0AW, United Kingdom; g Oxford Vaccine Group, Department of Paediatrics, University of Oxford, Oxford OX3 9DU, United Kingdom; h University of Antananarivo, Antananarivo, Madagascar; i Heidelberg Institute of Global Health, University of Heidelberg, Heidelberg, Germany; j UCLA Fielding School of Public Health, Los Angeles, CA 90095-1772, USA; k Health Economics Unit, University of Birmingham, Birmingham, United Kingdom

**Keywords:** Bangladesh, Cholera, Cost, Vaccine

## Abstract

Cholera poses a substantial health burden in the developing world due to both epidemic and endemic diseases. The World Health Organization recommends oral cholera vaccines for mass vaccination campaigns in addition to traditional prevention practices and treatments in resource-poor settings. In many developing countries like Bangladesh, the major challenge behind implementing mass vaccination campaigns concerns the affordability of the oral cholera vaccine (OCV). Vaccination of children with OCV is not only an impactful approach for controlling cholera at the population level and reducing childhood morbidity but is also considered more cost-effective than vaccinating all ages. The aim of the study was to estimate the cost of an OCV campaign for children from a societal perspective using empirical study. A total of 66,311 children aged 1 to 14 years old were fully vaccinated with two doses of the OCV Shanchol while 9,035 individuals received one dose of this vaccine. The estimated societal cost per individual for full vaccination was US$ 6.11, which includes the cost of vaccine delivery estimated at US$ 1.95. The cost per single dose was estimated at US$ 2.86. The total provider cost for full vaccination was estimated at US$ 6.01 and the recipient cost at US$ 0.10. Our estimation of OCV delivery costs for children was relatively higher than what was found in a similar mass OCV campaign for all age groups, indicating that there may be additional cost factors to consider in targeted vaccine campaigns. This analysis provides useful benchmarks for the possible costs related to delivery of OCV to children and future OCV cost-effectiveness models should factor in these possible cost disparities. Attempts to reduce the cost per dose are likely to have a greater impact on the cost of similar vaccination campaigns in many resource-poor settings.

## Highlights

• Estimation of the cost of an OCV campaign targeting children is important for policy implication.
• Vaccine price was the highest cost driver among the variable costs.
• Cost difference can be attributed to the scale and location of vaccination.

## 1 Introduction

Cholera is an acute diarrheal disease that is responsible for a substantial health burden in the developing world and is endemic in Asia and Africa [1]. Cholera is caused by the ingestion of toxigenic *Vibrio cholerae* and is closely linked to poverty, poor sanitation, and lack of safe water [2], [3]. The disease is a significant cause of mortality and morbidity, particularly in children [4], [5]. It is estimated that approximately 2.86 million cholera cases and 95,000 related deaths occur annually in endemic countries where 1.30 billion people are at risk [1]. A large part of these deaths occur in resource limited countries in sub-Saharan Africa and Asia where access to improved drinking water source remains a significant challenge [6]. Cholera is endemic in Bangladesh, which ranks among countries with the highest number of people at risk for cholera [1], [7]. A recent nationwide surveillance network found that cholera is pervasive throughout the country, with substantial heterogeneities within and between geographic areas [8].

WHO has prequalified several oral cholera vaccines (OCVs) and coordinates a Gavi-funded global stockpile of the OCV in 2013 managed by the International Coordinating Group (ICG) as well as by the Global Task Force on Cholera Control to ensure rapid access to vaccines for outbreak and humanitarian emergency situations, as well as control of cholera in endemic hotspots [5], [9], [10]. Different randomized trials conducted in a variety of settings (Bangladesh, India) have demonstrated that the OCV Shanchol confers substantial protective effectiveness, lasting for three to five years [11], [12], [13], [14]. The Expanded Program on Immunization (*EPI*) in Bangladesh is one of the most successful programs in the health sector in reducing childhood morbidity and mortality from vaccine preventable diseases [15]. The decision to introduce new vaccines into the *EPI* program should be supported by public health impact evaluations as well as economic analysis. It is crucial to estimate the cost of vaccination, including the cost of delivery and other sundry costs, for policymakers to prioritize effective nationwide vaccination programs. Although a number of studies have evaluated the cost of cholera vaccine delivery targeting all age groups, no studies have focused on campaigns targeted at only children [1], [2], [3], [4], [5], [6], [7]. In cholera endemic areas, OCV programs for children ages 1 to 14 years old are considerably more cost-effective than vaccination of all age groups [8]. The greater cost-effectiveness of vaccinating children is due to both the higher incidence rates among children and the herd immunity effects of adults by vaccinating children. The aim of the study was to estimate the cost of an OCV campaign targeting on children in a lower-middle income country using empirical data. Furthermore, identifying the relative contribution of different cost components to the total cost and estimating the cost per fully-vaccinated individuals were other objectives of this study. This analysis provides a comprehensive account of the cost components involved in deploying cholera vaccines to children in high-risk, urban areas.

## 2 Methodology

### 2.1 Study site

The study featured a mass vaccination campaign carried out in different administrative wards of Dhaka South City Corporation (DSCC), including Kamrangirchar, Hazaribag and part of Rayerbazar. These areas are densely populated while also lacking in adequate housing which further exacerbates the vulnerability to waterlogging and disease outbreaks associated with poor drainage and sanitation systems [16].

Social mobilization activities involved to inter-personal communication by the field workers and focal advocacy meetings in the catchment area. Prior to the vaccination campaign, a group of trained workers and volunteers visited each target site to distribute vaccination cards, and present messages related to cholera, OCV and vaccination activities. Communities were reminded by the volunteers about their vaccination date and time and to bring their ID card to the vaccination site. Targeted cell-phone messages and banners at vaccination sites were used to create awareness and encourage the participation in the vaccination program. Community leaders, both elected and appointed, were sought out and enlisted to help spread the word about the importance of immunization.

### 2.2 Vaccination campaign

The oral cholera vaccination program was conducted in the study areas with a two-dose regimen of Shanchol vaccine between January and February 2016. The single dose vials of Shanchol were stored at recommended storage temperature of 2–8 °C at *EPI* headquarters for this study. The target population was children aged 1 to 14 years old living in the urban communities. The first dose campaign was conducted from 23 January to 4 February, followed by the second dose from 6 February to 18 February. Before the vaccination campaign a broader baseline census was carried out to determine the target population in the study areas. A group of trained staff made house-to-house visits and collected demographic and social information of each household member. Unique household and member identification numbers were generated, and laminated identity (ID) cards were distributed to household members. This ID card contained household ID, member ID, individual name, age, and household location. Individuals were asked to show their study ID cards when receiving vaccine for verification and record-keeping during the program. All registered household members aged 1 to 14 years living in the study areas were invited to participate in the vaccination campaign.

To implement vaccine delivery, the program chose feasible sites such as school/college campuses, government/non-government institutions, ground floor/parking spaces of houses, and other open spaces in each study area, keeping in mind the maximum accessibility of the study population. Before and during the vaccination campaign, promotional activities in the form of media announcements, leaflets, and posters distribution were carried out. Field workers and volunteers also visited targeted households in selected areas to inform residents about the vaccination program and the time and place of vaccination. Local health facilities, pharmacies, and community residents were also involved to encourage attendance.

Informed written consent from guardians as well as assent from children aged 11–14 years were taken before vaccinating individuals. Each vaccination site was equipped with first aid boxes and drinking water for vaccine recipients. In case of any adverse event due to vaccination, a trained medical team was assigned to each vaccination site for emergency treatment. Participants who received the first dose of vaccine were eligible to receive 2nd dose of vaccine, with an interval of at least 14 days between doses in this study. After vaccinating, empty vials and aluminum foils were kept separate from other general waste in biohazard collecting waste bags. All unused vials in good condition from all vaccine sites were collected at the end of the day and restored to central cold storage for use the next day. To maintain the cold chain, packing, transportation and distribution of vaccine and logistics and waste management, 12 people were engaged throughout the period. Although the campaign essentially took place at a fixed location, certain teams functioned as mobile teams and others had different prices based on importance for the successful vaccination program.

### 2.3 Perspective of this study

Vaccination cost was estimated from societal perspective, which incorporates the costs from both the provider and household perspectives. Cost from societal perspective arises from three main elements: cost of acquiring the vaccines from the manufacturer, cost of vaccine delivery and administration, and cost incurred by the household to receive the vaccine [17]. Thus, the societal cost can be obtained by adding up the aforementioned three cost components.

### 2.4 Data collection

The study employed quantitative techniques to collect the cost data based on the financial records of purchasing, transporting, and administering the vaccine. Other related programmatic costs were also qualitatively gathered through document review, observational checklist, interviews with program managers, and related authorities. Vaccinated individuals also incur time and monetary costs for traveling to the vaccination sites, potentially queuing for the vaccine, and the time requirements associated with the vaccination protocol. We have included these costs as well.

### 2.5 Data analysis

All resource items used for vaccine delivery activities were captured using an “ingredients” approach of micro-costing. In the ingredients approach, all types of inputs were listed with their respective quantities and costs by activity [18]. Fixed and variable costs were captured through a comprehensive list of activities during the time of vaccination. Fixed costs were those which were essential for setting up and running the vaccination campaign and do not vary with the number of vaccinated people. Variable costs, on the other hand, vary with the number of people being vaccinated. The major activities of this mass vaccination campaign were in vaccine procurement from the manufacturer, storage and cold chain management, training, social mobilization, vaccine delivery to the selected population, and waste management. During this campaign, cold chain management related activities were performed at no cost by the Government of Bangladesh. Although actual expenditure for these items were zero, the shadow prices for each item were obtained from the market prices and included in our analysis.

Prices of all capital items such as vaccine cold boxes, vaccine carriers, and dial thermometers were discounted using an inflation adjusted rate and annualized using their respective functional lifetime to calculate the allocated cost for the program. For this purpose, we applied a 3 % discounting rate [17]. This rate was then adjusted according to the average inflation rate, which was 7.01 % for the period 2011–2015 as reported by the Bangladesh Central Bank. Shared cost items (cold chain storage, refrigerator) were apportioned according to the proportion of time usage of the relevant item or activity. The rental prices of vehicles used during the vaccination campaign were also included in the analysis, apportioned in a similar approach.

The monetary value of time spent by some senior management staff of vaccine project were obtained after discussion with program management team and added to our estimation by calculating each staff member’s time involvement in the study and their respective salaries.

To obtain the indirect costs incurred by the vaccine recipients, such as the travel and waiting time taken to receive the vaccine, we used an age-specific human capital approach. For this calculation, vaccine recipients were categorized into two age groups: children < 5 years of age, and children between 5 and 14 years of age. We considered the cost of travel and waiting time as zero for the younger group. For the older age group, the cost of time lost by vaccine recipients and attendants (e.g., parents, guardians) was calculated by generating per minute income of the attendants, and multiplying that measure by the time spent for vaccination. When income of the attendant was not available, such as for those not directly involved in economic activities, the monthly income was replaced by the minimum wage rate of Bangladesh.

Sensitivity analyses were conducted to determine the range of cost estimates for different scenarios pertaining to delivery activities. We examined the effect of changes in the price of vaccines and the salary levels of the staff; salary level was of interest because relatively lower salary levels may be more appropriate for Bangladesh rather than that of the icddr,b project staff. A univariate analyses was conducted to observe the possible effects of the main cost drivers on the cost of full vaccination:

$$Y=\beta +\left(\frac{\mathrm{aSMc}+bVc+cSLc+dRVc+ePc}{N}\right)$$

Where, *Y* = per patient fully vaccination cost, *β* = constant = 0.651 (calculated for vaccination cost), SMc = social mobilization cost, Vc = vaccine price, SLc = salary cost, RVc = recipients cost for vaccination, *Pc* = printing cost, N = number of fully vaccinated individuals; a, b, c, d, e is the percentage changed (initially 100 %) for social mobilization, vaccine price, salary cost, recipients vaccination cost and printing cost respectively in sensitivity analyses. All costs were converted into US dollars (US$) using the average official government exchange rate of 78.25 Bangladeshi Taka (BDT) to US$ 1.

## 3 Results

**Fig. 1:**
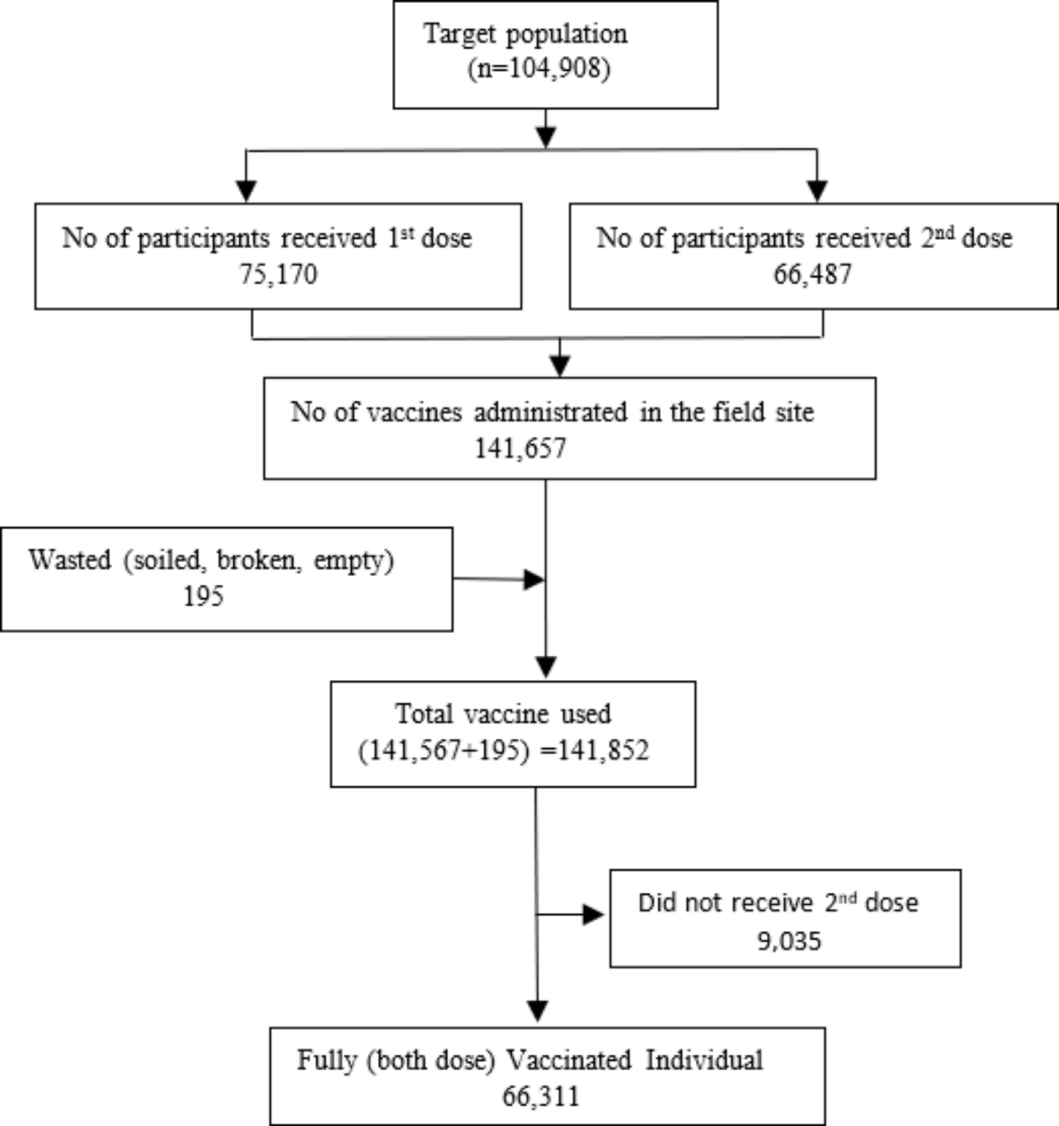
Target population and Oral Cholera Vaccine (OCV) delivery status during vaccination campaign.

During the vaccination campaign, a total of 141,852 doses of vaccine were used, with 75,170 administered as the 1st dose and 66,487 administered as the 2nd dose; 195 vaccine vials were wasted as they were broken, soiled or damaged. In total 66,311 individuals received full vaccination (both 1st and 2nd dose) and the remaining 9,035 individuals received incomplete vaccination (not received 2nd dose) (Fig. 1). The dropout rate between the 1st and 2nd round was 12.02 %.

(Fig. 1 will be inserted here).

### 3.1 Cost of vaccination program

**Fig. 2:**
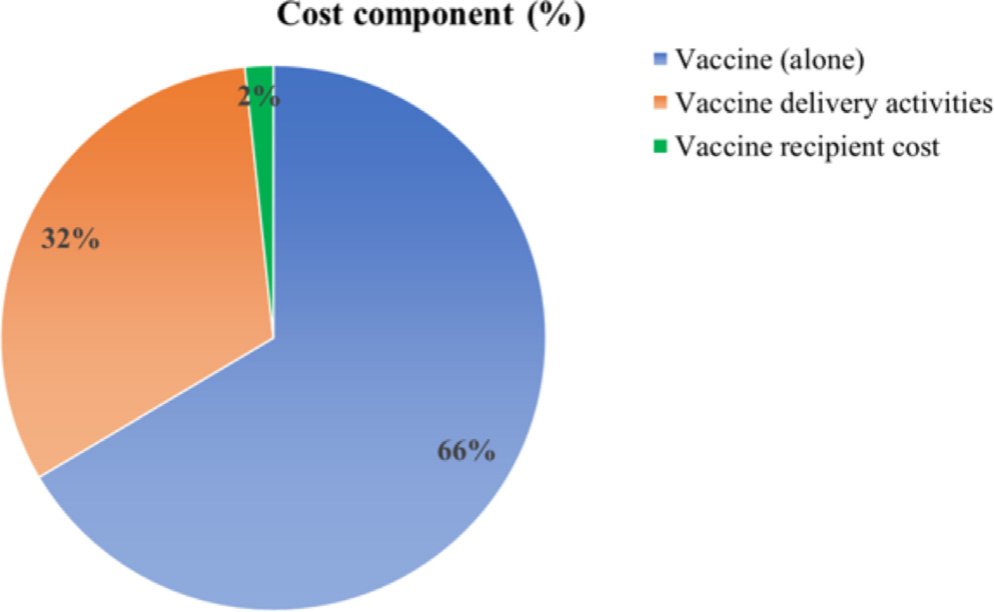
Distribution of cost components (%).

The total cost of the vaccination campaign was US$ 405,445. Fig. 2 showed the vaccine procurement cost was US$ 269,519 which included freight charges, insurance, international and domestic transport. The vaccine delivery-related cost was US$ 129,159 and the cost incurred by the vaccine recipient was US$ 6,767 (Fig. 2).

Fixed costs accounted for 1.9 % of the total cost of vaccination, while variable costs accounted for 98.08 %. Considering all cost components, vaccine price was the main cost driver, constituting 66.47 % of the total cost, followed by staff salaries which comprised of 18.09 % of total cost. Fixed costs of the vaccination campaign amounted to US$ 7,804, and the cost of social mobilization and promotions was the largest cost component within the fixed costs.at US$5,787 Social mobilization activities featured a variety of advocacy meetings arranged before and throughout the vaccination campaign, including meetings with local government representatives, NGOs, and pediatric associations. The cost of social mobilization and promotion included the cost of promotional banners, leaflets, meeting-related cost as per-diem, and other promotional activities. Moreover, six different long-term training sessions were conducted with the supervisors and volunteers for demonstrating the vaccine administration. For this purpose, US$ 175 was spent for training-related activities.

Regarding the total cost distribution, it was found that vaccine price was the highest cost driver among the variable costs and also among total vaccination campaign cost. This cost component accounted for 66.47 % of total costs. Vaccine vials were imported at a special rate of US$ 1.85, and after adjusting the freight charges, insurance, international and domestic transport, finally the cost per vial was calculated as US$ 1.90. A total of 141,852 vaccine doses were used in the vaccine sites, yielding US$ 269,519 for vaccine costs. The estimated societal cost per patient for full vaccination was US$ 6.11, among which the delivery cost was US$ 1.95. Total cost for each dose of vaccine was US$ 2.86 including US$ 0.91 for delivery cost. The total provider cost for full vaccination was US$ 6.01 and the recipient cost was US$ 0.10 which in together yielded the societal cost.

### 3.2 Cold chain management and designing stationaries

Cold chain-related costs were another important contributor to fixed costs. When the vaccines were shipped to Bangladesh, the vials were temporarily stored at the central *EPI* cold-storage and later sent to field sites on demand. A total of US$ 1,164 was spent for cold-chain and management-related activities, including the costs of cold boxes, vaccine carriers, thermometers, and cold storage at field sites. Additionally, a total of US$ 678 was used to develop the micro plan guide for the vaccination campaign, outline strategic guidelines, and produce other manuals for managers, supervisors, those administering the vaccine, and volunteers. This cost also covered the capacity building of human resources, advocacy, communication and social mobilization, program support costs, recruitment of health professionals, community health workers, and volunteers, supporting the creation and scaling up of new performance-based incentives systems, reward/incentive payments to health workers, volunteers, or community health workers.

### 3.3 Staff salaries, transportation, and other variable costs

During the mass vaccination campaign, a total of US$ 73,329 was paid as honoraria to all staff related to the vaccination program, including personnel salaries and communication allowances for icddr,b staff members and small allowances for non-staff personnel such as cold chain packers, supervisors, and volunteers. In total, 70 vaccinators, 70 volunteers, 70 recordkeepers, 40 Tab operators and 20 first-line supervisors were engaged in vaccine delivery. A total of 25 study staff as well as investigators, physicians, and other staff acted as second-line supervisors. However, the investigator and second-line supervisors were involved in the training of the trainers and training of the vaccine delivery staff as well as monitoring the campaign. To maintain the cold chain, packing, transportation, and distribution of vaccines and logistics, 12 people were engaged throughout the period. All staffs were trained adequately on the vaccination program and their responsibilities. This cost component was 18.09 % of the total costs of the vaccination program.

Transportation costs throughout the vaccination campaign totaled US$ 4,675; transportation included carriage of vaccines, waste, refreshment food, and other logistic materials. Printing costs totaled US$ 6,866, and included costs for printing participant consent forms, study master lists, and census forms. The cost of identification cards distributed to the study population for implementing the campaign came to US$ 1,476. Stationary costs totaled US$ 4,361 for purchase of items such as pens, pads, pencils, forceps, and other non-stock items used in vaccination field sites and the administrative office. Refreshments at vaccination sites cost US$ 20,779 and included water jars, disposable glasses, water, and food. Finally, for decorating the vaccination sites, US$ 5,087 was spent on tents, tables, and chairs.

After administrating the vaccines, empty vials and other supplies were disposed of according to local guidelines. A total of US$ 3,185 was spent on waste management, including US$ 1,332 for incineration and US$ 1,853 for the purchase of biohazard bags and waste-baskets.

### 3.4 Recipient cost for vaccination

The total cost incurred by participants to receive the vaccine was estimated to be US$ 6,767. This cost included all direct costs to the participants such as travel and food, and indirect costs such as time lost by participants to receive the vaccine, including time for travel and attendance.

### 3.5 Sensitivity analysis

**Fig. 3:**
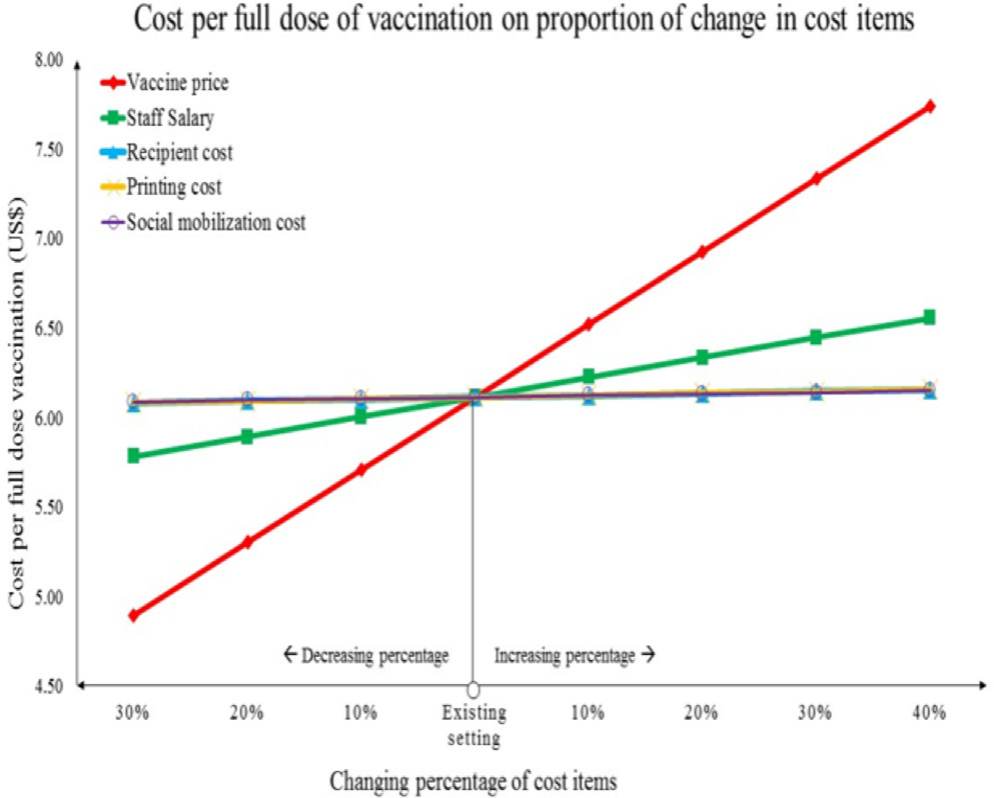
Effect of changing percentage of selected cost items on full dose vaccination cost.

To assess the impact of key cost components like vaccine price, staff salary, social mobilization cost, recipients cost for vaccination, and printing cost on the overall cost of full dose vaccination, a univariate sensitivity analysis was performed. In Fig. 3 lines representing different cost components demonstrate their effects on full dose vaccination cost based on percentage change of estimated costs, where greater sloped lines show higher responses. Initially, at the existing set point with the true estimated cost of items (Table 1), the cost of a full regimen of vaccination was US$ 6.11. A 10 % increment in the vaccine price came to US$ 6.52. In this scenario, the total cost per fully vaccinated person would be increased by 6.65 %. On the contrary, 20 % decrease in staff salary would make the cost per fully vaccinated individual US$ 5.89 with a 3.62 % decrease from initial cost (see Table 2).

**Table 1:** Distribution and proportion of cost items for vaccine administration.

Cost Categories	Cost Components	Cost (USD)	Percentage
Fixed Cost	Designing Stationaries	678	0.17
	Social Mobilization and Campaign	5,787	1.43
	Long term training	175	0.04
	Cold chain related	1,164	0.29
*Total fixed cost*		7,804	1.92
Variable Cost	Vaccine	269,519	66.47
	Transportation	4,675	1.15
	Salaries and communications	73,329	18.09
	CVIS Card	1,476	0.36
	Decoration	5,087	1.25
	Rent	1,597	0.39
	Vaccine recipient cost	6,767	1.67
	Refreshment	20,779	5.12
	Stationaries (Pen, pencil, forceps etc)	4,361	1.08
	Printing	6,866	1.69
	Waste Management	3,185	0.79
*Total variable cost*		397,641	98.08
**Total Cost**		**405,445**	**100.00**

**Table 2:** Per patient cost of vaccination.

**Cost description**	**Per patient cost (US$)**
Total cost for full dose vaccination	6.11
Total cost for single dose vaccination	2.86
Vaccine delivery cost for full dose ^a^	1.95
Vaccine delivery cost for single dose ^a^	0.91
Total provider cost for full dose vaccination	6.01
Total recipient cost for full dose vaccination	0.10
**Societal cost**^b^	**6.11**
^a^ Excluding vaccine price, ^b^ Sum of provider cost and recipient cost	

## 4 Discussion

This analysis is the first empirical evaluation of OCV costs in a program exclusively targeting children. We estimated the total societal cost of delivering a two-dose regimen of OCV to children living in urban communities by comprehensively costing a mass vaccination campaign that reached 75,346 children in Dhaka, Bangladesh.

With the increased interest in controlling cholera through vaccination, research to investigate and analyze the cost of immunization programs has been gaining traction. While a number of analyses on the delivery cost of OCV have been published, few of these reports feature sufficient detail regarding the data collection of cost components, and none are focused on delivery only to children [19], [20], [21], [22].

Our comprehensive evaluation of OCV costs took into account the indirect costs of vaccination, such as the loss of income and transportation costs of vaccine recipients, which are critical elements that are often overlooked in other cost estimation research. We assessed these indirect costs using an age-specific human capital approach, which fully considered the lost time and income of vaccine recipients and their adult attendants. Incorporating these cost dimensions is crucial for a better understanding of the total costs of vaccination from a broader societal perspective [19]. In our study we have intended to explore the distribution and cost drivers for a large-scale vaccination intervention. We assumed that the vaccine does not cause any adverse effects causing treatment cost, which is supported by evidence from the previous trials [23], [24].

There are some limitations of this study. Firstly, the study vaccination campaign was conducted in a few selected areas within Dhaka. As such, the cost of vaccination may vary if the vaccination campaign were conducted in different geographic areas or during different seasons. Furthermore, foregone time due to vaccination could not be exactly valued for school-aged children, leading to imprecision regardless of the method used to value their time. There may also be some reporting bias in the financing data, which were extracted from a variety of sources documents and interviewing the respective authorities. Despite these limitations, the analysis demonstrates the evaluation of the societal cost of the cholera vaccination program using empirical data. The findings of this analysis are encouraging and also demonstrate a methodological approach for estimating costs which can be applied even in rural Bangladesh as well as in the routine public health practice.

Overall, the cost estimations found in this analysis are consistent with those determined in other studies of OCV delivery, with the crucial caveat that these studies feature OCV programs aimed at all age groups rather than exclusively children [19], [20], [21], [22]. Various modeling-based studies indicated that vaccination strategy targeting children appeared more cost-effective than a strategy that included vaccinating adults due to having higher incidence rates of endemic cholera in children than in adults [25], [26], [27], [28]. However, none of the studies analysed the cost of vaccination for children, such as the empirical data obtained in this study. Several studies indicated that low-cost vaccines often most cost-effective strategy for controlling infectious diseases, especially in resource poor countries like Bangladesh [29], [30]. Similar to other studies we also observed that vaccine procurement price was the largest cost driver (66 % of total cost) [21], [31]. The second largest cost driver found in our analysis was staff salary (18 %). Although the campaign essentially took place at a fixed location, certain teams functioned as mobile teams to vaccinate the persons who did not present for vaccination at fixed sites. This cost driver was also found as the second highest contributor in another study in Haiti, which found that salary accounted for 14 % of total cost [32]. The relatively higher proportion of staff cost in this present study can be attributed to the relatively high staff salary level at icddr,b compared to that of government employees who typically conduct such mass immunization programs.

This study observed that the cost per single dose of OCV vaccination was US$ 2.86 while the vaccine delivery cost was US$ 0.91 which was relatively lower than the vaccination campaign in Malawi where total economic cost per partially immunized person was US$5.43 [21]. In a study in Haiti observed the estimated per dose vaccination cost was US$ 2.90 including US$ 0.70 for vaccine delivery cost using Shanchol vaccine [32]. In a 2-dose OCV vaccination campaign with Dukoral in an urban setting in Mozambique, the estimated delivery cost was US$ 2.09 per fully vaccinated person which was a more expensive vaccine [33]. This is similar to our calculated delivery cost of US$ 1.95 per fully vaccinated person. Moreover, the delivery costs of OCV through mass vaccination campaign might differ depending on the local context and geographical terrain as well as the availability of vaccine delivery infrastructure. For instance, delivery cost of Shanchol was estimated US$ 1.14 in India and US$ 3.05 in South Sudan per fully vaccinated person [19]. This cost difference could be partially attributed to the scale of vaccination and nature of the health care setting where vaccination was conducted.

Regarding vaccination cost using Shanchol vaccine, we found that the societal cost per fully vaccinated individual was estimated at US$ 6.11 which was higher than what was found in a previous study conducted in urban Bangladesh [20]. This differential is largely due to the significantly different study contexts and target population of the vaccination campaigns: in the earlier vaccination campaign, all high-risk people irrespective of age were targeted and approximately 123,661 were fully vaccinated, totaling almost twice as many vaccine recipients than in the current study, in which only young children up to 14 years old were included [20]. However, if the total quantity/output (here, vaccine recipient) is higher, the average cost will be lower in short run. We observed that the cost for a complete two-dose regimen was higher than the estimated cost from adding together the costs of two single dose regimens (US$ 6.11 vs US$ 5.72 (2.86 × 2)). This disparity occurred due to the additional cost occurred from incomplete doses (either only 1st dose or only 2nd dose).

Overall, the cost of full oral cholera vaccination estimated in this analysis was relatively similar to the costs reported from Shanchol OCV campaigns that target all age groups. The cost per fully vaccinated person of a two-dose OCV campaign in Haiti was US$ 5.8 excluding the household cost [32]. Similarly, an Ethiopian study they estimated that the cost was US$ 5.85 per fully vaccinated person [34]. In the current analysis the estimated fixed cost was only 1.92 % of total cost and as this fixed cost was not extensive, the cost per person fully vaccination may vary by sample size and vaccine coverage rate.

There are several limitations as the estimation was performed using the costs incurred during the vaccination program at this time and we did not analyze the impact of inflation or indeed reduction in vaccine costs in the near future. Again, we did not analyze the cost of vaccination if the vaccine coverage rate may have varied or if the vaccination campaign had been conducted in outside the Dhaka or even other geographical location. We did not include the cost of census-related activities as the coverage was estimated from the data already available in the OCV project. Further there is a limitation of generalizing this costing exercise to other settings in Sub-Saharan Africa and/or humanitarian settings where the OCV campaign is very common.

## 5 Conclusion

In this analysis, we found that the total societal cost was relatively greater in an OCV campaign exclusive to children compared to ones reported elsewhere for all ages. While this higher cost may be unexpected, it should also be noted that vaccinating children with OCV may have greater impact on cholera than vaccinating adults. Multiple cost-effectiveness models have found that vaccinating children, rather than all age-groups, is more cost-effective in cholera endemic countries [26]. Vaccinating children with OCV is of special interest to policymakers, and greater research is needed to fully elucidate the empiric costs and gains from child-focused OCV strategies.

## Declaration of Competing Interest

The authors declare that they have no known competing financial interests or personal relationships that could have appeared to influence the work reported in this paper.

## Data Availability

Data will be made available on request.

## References

[1] Ali M, Nelson AR, Lopez AL, Sack DA. Updated global burden of cholera in endemic countries. PLoS Negl Trop Dis. 2015;9: 1–13. doi:10.1371/journal.pntd.0003832. 10.1371/journal.pntd.0003832 PMC4455997 26043000

[2] World Health Organization. Weekly epidemiological record. Cholera vaccine: WHO position paper. Geneva 27, Switzerland; Mar 201085: 117–128. doi:10.1186/1750-9378-2-15.

[3] World Health Organization. Cholera outbreak: assessing the outbreak response and improving preparedness: Global Task Force on Cholera Control. 2004.

[4] Mokomane M., Kasvosve I., de Melo E., Pernica J.M., Goldfarb D.M. (2018). The global problem of childhood diarrhoeal diseases: emerging strategies in prevention and management. Ther Adv Infect Dis.

[5] World Health Organization (WHO). Cholera vaccines: WHO position paper. Weekly Epidemiological Record. Geneva 27, Switzerland; Aug 201734: 477–500. doi:10.1371/jour.

[6] Unicef, WHO. Progress on Sanitation and Drinking Water: 2015 Update and MDG Assessment. 2015. doi:10.1007/s13398-014-0173-7.2.

[7] Qadri F., Ali M., Chowdhury F., Khan A.I., Saha A., Khan I.A. (2015). Feasibility and effectiveness of oral cholera vaccine in an urban endemic setting in Bangladesh: A cluster randomised open-label trial. Lancet.

[8] Khan A.I., Rashid M.M., Islam M.T., Afrad M.H., Salimuzzaman M., Hegde S.T. (2020). Epidemiology of cholera in bangladesh: Findings from nationwide hospital-based surveillance, 2014–2018. Clin Infect Dis.

[9] HERA. External evaluation of the international coordinating group on vaccine provision (ICG). Belgium; 2017.

[10] Wierzba T.F. (2019). Oral cholera vaccines and their impact on the global burden of disease. Hum Vaccin Immunother.

[11] Bhattacharya S.K., Sur D., Ali M., Kanungo S., You Y.A., Manna B. (2013). 5 year efficacy of a bivalent killed whole-cell oral cholera vaccine in Kolkata, India: A cluster-randomised, double-blind, placebo-controlled trial. Lancet Infect Dis Elsevier Ltd.

[12] Islam MT, Chowdhury F, Qadri F, Sur D, Ganguly NK. Trials of the killed oral cholera vaccine (Shanchol) in India and Bangladesh: Lessons learned and way forward. Vaccine. 2020. doi:10.1016/j.vaccine.2019.06.082. 10.1016/j.vaccine.2019.06.082 31301917

[13] Bi Q., Ferreras E., Pezzoli L., Legros D., Ivers L.C., Date K. (2017). Protection against cholera from killed whole-cell oral cholera vaccines: a systematic review and meta-analysis. Lancet Infect Dis.

[14] Ali M., Qadri F., Kim D.R., Islam M.T., Im J., Ahmmed F. (2021). Effectiveness of a killed whole-cell oral cholera vaccine in Bangladesh: further follow-up of a cluster-randomised trial. Lancet Infect Dis.

[15] Ministry of Health and Family Welfare Government of the People’s Republic of Bangl;adesh. National Immunization Policy. Dhaka, Bangladesh; 2013.

[16] Adams A.M., Islam R., Ahmed T. (2015). Who serves the urban poor? A geospatial and descriptive analysis of health services in slum settlements in Dhaka. Bangladesh Health Policy Plan.

[17] Cook J., Jeuland M., Whittington D., Poulos C., Clemens J., Sur D. (2008). The cost-effectiveness of typhoid Vi vaccination programs: Calculations for four urban sites in four Asian countries. Vaccine.

[18] Drummond M, Sculpher MJ, Laxton KC, Stoddart GL, Torrance GW. Methods for the Economic Evaluation of Health Care Programmes. Third edit. Oxford University Press; 2005.

[19] Mogasale V, Ramani E, Wee H, Kim JH. Oral Cholera Vaccination Delivery Cost in Low- and Middle-Income Countries: An Analysis Based on Systematic Review. PLoS Negl Trop Dis. 2016;10: 1–15. doi:10.1371/journal.pntd.0005124. 10.1371/journal.pntd.0005124 PMC5145138 27930668

[20] Sarker A.R., Islam Z., Khan I.A., Saha A., Chowdhury F., Khan A.I. (2015). Estimating the cost of cholera-vaccine delivery from the societal point of view: A case of introduction of cholera vaccine in Bangladesh. Vaccine Elsevier Ltd.

[21] Ilboudo P.G., Le Gargasson J.-B. (2017). Delivery cost analysis of a reactive mass cholera vaccination campaign: a case study of Shanchol^TM^ vaccine use in Lake Chilwa, Malawi. BMC Infect Dis. BMC Infect Dis.

[22] Mogasale V, Kar SK, Kim JH, Mogasale V V., Kerketta AS, Patnaik B, et al. An Estimation of Private Household Costs to Receive Free Oral Cholera Vaccine in Odisha, India. PLoS Negl Trop Dis. 2015;9. doi:10.1371/journal.pntd.0004072. 10.1371/journal.pntd.0004072 PMC4564266 26352143

[23] Clemens J., Harris J., Khan M.R., Kay B., Yunus M., Svennerholm A.-M. (1986). Field Trial of Oral Cholera Vaccines in Bangladesh. Lancet.

[24] Naficy A.B., Trach D.D., Ke N.T., Chuc N.T.K., Sorkin A., Rao M.R. (2001). Costs of immunization with a locally-produced, oral cholera vaccine in Vietnam. Vaccine.

[25] Dimitrov DT, Troeger C, Halloran ME, Longini IM, Chao DL. Comparative Effectiveness of Different Strategies of Oral Cholera Vaccination in Bangladesh: A Modeling Study. PLoS Negl Trop Dis. 2014;8: 1–8. doi:10.1371/journal.pntd.0003343. 10.1371/journal.pntd.0003343 PMC4256212 25473851

[26] Ivi (2012). An Investment Case for the Accelerated Introduction of Oral Cholera Vaccines. Seoul, South Korea.

[27] Troeger C., Sack D.A., Chao D.L. (2014). Evaluation of Targeted Mass Cholera Vaccination Strategies in Bangladesh : A Demonstration of a New Cost-Effectiveness Calculator. Am J Trop Med Hyg.

[28] Khan AI, Levin A, Chao DL, DeRoeck D, Dimitrov DT, Khan JAM, et al. The impact and cost-effectiveness of controlling cholera through the use of oral cholera vaccines in urban Bangladesh: A disease modeling and economic analysis. PLoS Negl Trop Dis. 2018;12: e0006652. doi:10.1371/journal.pntd.0006652. 10.1371/journal.pntd.0006652 PMC6177119 30300420

[29] Jeuland M., Cook J., Poulos C., Clemens J., Whittington D. (2009). Cost-effectiveness of new-generation oral cholera vaccines: A multisite analysis. Value Heal.

[30] Russell L.B., Sobanjo-ter Meulen A., Toscano C.M. (2021). Evaluating the cost-effectiveness of maternal pertussis immunization in low- and middle-income countries: A review of lessons learnt. Vaccine.

[31] Ngabo F., Levin A., Wang S.A., Gatera M., Rugambwa C., Kayonga C. (2015). A cost comparison of introducing and delivering pneumococcal, rotavirus and human papillomavirus vaccines in Rwanda. Vaccine Elsevier Ltd.

[32] Routh J.A., Sreenivasan N., Adhikari B.B., Andrecy L.L., Bernateau M., Abimbola T. (2017). Cost Evaluation of a Government-Conducted Oral Cholera Vaccination Campaign—Haiti, 2013. Am J Trop Med Hyg.

[33] Cavailler P., Lucas M., Perroud V., Mcchesney M., Ampuero S., Guerin P. (2006). Feasibility of a mass vaccination campaign using a two-dose oral cholera vaccine in an urban cholera-endemic setting in Mozambique. Vaccine.

[34] Teshome S., Desai S., Kim J.H., Belay D., Mogasale V. (2018). Feasibility and costs of a targeted cholera vaccination campaign in Ethiopia. Hum Vaccin Immunother Taylor & Francis.

